# SIRT5 Deficiency Enhances Susceptibility to Kainate-Induced Seizures and Exacerbates Hippocampal Neurodegeneration not through Mitochondrial Antioxidant Enzyme SOD2

**DOI:** 10.3389/fncel.2016.00171

**Published:** 2016-06-27

**Authors:** Fengling Li, Lei Liu

**Affiliations:** ^1^Department of Pharmacy, Linyi Tumor HospitalLinyi, Shandong, China; ^2^Department of Anesthesiology, University of FloridaGainesville, FL, USA; ^3^Center for Translational Research in Neurodegenerative Disease, University of FloridaGainesville, FL, USA

**Keywords:** Sirtuin, SIRT5, mitochondria, oxidative stress, kainate, seizures, reactive astrogliosis, SOD2

## Abstract

Epilepsy is a common and serious neurological disorder characterized by occurrence of recurrent spontaneous seizures, and emerging evidences support the association of mitochondrial dysfunction with epilepsy. Sirtuin 5 (SIRT5), localized in mitochondrial matrix, has been considered as an important functional modulator of mitochondria that contributes to ageing and neurological diseases. Our data shows that SIRT5 deficiency strikingly increased mortality rate and severity of response to epileptic seizures, dramatically exacerbated hippocampal neuronal loss and degeneration in mice exposed to Kainate (KA), and triggered more severe reactive astrogliosis. We found that the expression of mitochondrial SIRT5 of injured hippocampus was relatively up-regulated, indicating its potential contribution to the comparably increased survival of these cells and its possible neuroprotective role. Unexpectedly, SIRT5 seems not to apparently alter the decline of antioxidant enzymes superoxide dismutase 2 (SOD2) and glutathione peroxidase (GPx) in hippocampus caused by KA exposure in our paradigm, which indicates the protective role of SIRT5 on seizures and cellular degeneration might through different regulatory mechanism that would be explored in the future. In the present study, we provided strong evidences for the first time to demonstrate the association between SIRT5 and epilepsy, which offers a new understanding of the roles of SIRT5 in mitochondrial functional regulation. The neuroprotection of SIRT5 in KA-induced epileptic seizure and neurodegeneration will improve our current knowledge of the nature of SIRT5 in central nervous system (CNS) and neurological diseases.

## Introduction

Epilepsy is a common and serious neurological disorder that affects about 50 million people worldwide, characterized by the unpredictable but recurrent occurrence of spontaneous seizures (Pitkanen and Lukasiuk, 2011). The cellular and molecular mechanisms of seizures and epileptogenesis remain unknown. Recently, increasing evidences have been supporting the association of mitochondrial dysfunction and oxidative stress with epilepsy, not only as a consequence of epileptic seizures, but also as a contributor in epileptogenesis (Kunz, 2002; Patel, 2004; Waldbaum and Patel, 2010a). Kainate (KA), an analog of glutamate, is a 30-fold more potent neurotoxicant; and KA exerts its neuro-excitotoxic and epileptogenic effects by acting on glutamate receptors (Zaczek et al., 1981; Ferkany et al., 1984). The KA-induced seizure model is widely used as a model of human temporal lobe epilepsy (Ben-Ari and Cossart, 2000).

Mitochondrial dysfunction and oxidative stress have been implicated in the pathogenesis of many neurodegenerative diseases; however, their contribution to the development of epilepsy is still unclear (Waldbaum and Patel, 2010b; Rowley and Patel, 2013). Mitochondria have various pivotal cellular functions including production of ATP, regulation of intracellular Ca^2+^ signaling, and production of reactive oxygen species (ROS), critically affecting neuronal excitability. Functional defect of mitochondria has been observed in human and animal epilepsy (Kunz et al., 2000). For example, mitochondrial oxidative stress has been demonstrated to be a contributing mechanism to epilepsy in mitochondrial antioxidant (Sod2^−/−^) deficient mice that exhibited spontaneous electrographic and motor seizures (Liang et al., 2012). Experimental epileptic seizures have been reported to cause acute oxidative damage to cellular macromolecules that ultimately leads to neuronal death in susceptible brain regions, but long-term effect of seizures on mitochondrial defect and oxidative stress is still obscure (Kudin et al., 2002).

Sirtuins are the family of nicotinamide adenine dinucleotide (NAD^+^)-dependent histone deacetylases that modulate distinct metabolic pathways, diverse stress responses and other biological activities in metabolism, aging and other diseases (Michishita et al., 2005). Sirtuin 5 (SIRT5), localized in mitochondrial matrix, is mostly expressed in brain, heart, liver, muscle and kidney (Mahlknecht et al., 2006; Schlicker et al., 2008). Previous studies showed that SIRT5 regulates the urea cycle by deacetylating and activating carbamoyl phosphate synthase 1 (CPS1; Nakagawa et al., 2009; Parihar et al., 2015). In addition to its deacetylase activity, SIRT5 exhibits demalonylase and desuccinylase activities (Du et al., 2011; Peng et al., 2011). The effect of SIRT5 on mitochondrial metabolism and function implies the potential positive correlation between SIRT5 and epilepsy. However, till today, the effect and mechanism of SIRT5 in the regulation of mitochondrial biology, such as energy production, metabolism and intracellular signaling in healthy brain and neurological diseases are still poorly understood. No report demonstrated the association of SIRT5 with epilepsy and the contribution of SIRT5 in the pathogenesis of seizure and of epilepsy.

Given the important role of SIRT5 in the functional regulation of mitochondria and our previous study, we hypothesized that SIRT5 is an important regulator in KA-induced neurodegeneration and the underlying oxidative stress. Accordingly, our study had three goals: (1) to determine whether the lack of SIRT5 can increase the seizure susceptibility and mortality by comparing SIRT5^−/−^ and wild-type (WT) mice exposed to KA; (2) to assess the effect of SIRT5 deficiency on hippocampal neurodegeneration and associated reactive astrogliosis; (3) to test whether KA exposure can alter SIRT5 expression and thereby affect mitochondrial antioxidant enzyme superoxide dismutase 2 (SOD2) and glutathione peroxidase (GPx) levels, which might be involved in the underlying mechanism of above neuroprotection. The present study will provide the evidence for the important role of SIRT5 on KA-induced seizure and epilepsy by using genetic ablation of SIRT5 in mice. Further investigation is needed to better understand the underlying mechanisms.

## Materials and Methods

### Animals

Eight to 12 weeks old male mice were housed on a 12 h light/dark cycle and allowed *ad libitum* access to food and water. SIRT5^−/−^ mice (*n* = 42) were purchased from Jackson laboratory. Age matched WT male C57BL/6 mice (*n* = 38) were used as controls. All animal procedures were performed in accordance with National Institute of Health guidelines and approved by Institutional Animal Care and the relevant Institutional Ethical Review Committees.

### Kainate Induced Seizure

Systemic injection of KA was used to investigate whether SIRT5 deficiency affected the behavioral seizures and the mortality rate. KA (Sigma, St. Louis, MO, USA) solutions were freshly prepared before use according to the manufacturer’s instruction. KA was dissolved at a concentration 2 μg/μl in alkalized isotonic saline. Adult mice were injected intraperitoneally with a single dosage (either 20 mg/kg or a higher dose of 25 mg/kg) of KA. KA-treated mice were evaluated every 20 min for 2 h after KA injection by trained experimenter blind to genotype. The behavioral seizures were scored 0–5 according to reference descriptions (Ben-Ari et al., 1981; Yang et al., 1997): 0, normal behavior; 1, immobilization, “wet-dog shakes” occasionally; 2, head nodding, unilateral forelimb clonus, frequent “wet dog shaking”; 3, rearing, bilateral forelimb clonus; 4, generalized limbic seizures with falling and running; 5, continuous generalized seizures with tonic limbic extension, death. The survival rate was analyzed 24 h after KA injection.

In addition to recording seizure scores, the severity of seizures was calculated by integrating individual scores of each mouse over the experiment period (Giménez-Cassina et al., 2012). This method incorporates the seizure severity of mice that died in 2 h following KA injection. Briefly, all scores of same mouse were added together and then divided by the total experimental time by using the formula: Seizure Severity each mouse = ∑ (all scores of each mouse)/experimental time. The mean value of the seizure severity in WT mice group was set as 100%, which was applied to normalize the value of tested genotype within the same scale.

### Histology and Immunohistochemistry

For preparation of brain sections, mice treated with KA (20 mg/kg, 2 mg/ml) or Saline were deeply anesthetized with isoflurane and sacrificed, then perfused transcardially with phosphate buffered saline (PBS, 150 mM NaCl, 10 mM Na-phosphate buffer, pH 7.4) followed by freshly prepared 4% paraformaldehyde in 0.1 M sodium phosphate buffer, 0.1 M phosphate buffer (pH 7.4). Brains were removed, postfixed overnight, gradiently dehydrated with 20 and 30% sucrose in PBS and frozen at −80°C. For each animal, six sets of 30-μm-thick coronal brain sections were cut on Leica cryostat and preserved in PBS-buffered 50% glycerol at −20°C until used for subsequent free-floating immunohistochemistry.

Sections were incubated in 3% H_2_O_2_ for 15 min at room temperature, and rinsed three times with PBS. After blocking in PBS containing 0.3% Triton X-100 and 5% bovine serum albumin for 1 h, the sections were then incubated with primary antibody overnight at 4°C and followed by incubation with appropriate secondary antibodies (Vectastain laboratories) in room temperature. Markers were visualized with 3,3-diaminobenzidine (DAB), sections were dehydrated and cover slipped with DPX mountant. Mouse anti-glial fibrillary acidic protein (GFAP) antibody (EMD Millipore, 1:2000) was used for staining in this study. The total number of GFAP-positive astrocytes in CA1 or CA3 region was counted. We also counted the percentage of GFAP-positive reactive astrocyte, characterized by hypertrophic cell bodies and intensely stained processes. Three consecutive sections per brain were analyzed and provided a single value for each mouse. The images were captured and analyzed by NIS-Elements AR microscope software and ImageJ software (NIH).

Neuronal death and degeneration were detected by Nissl (Cresyl Violet) and Fluoro-Jade C (FJc) staining. Nissl staining was performed in terms of the manufacturer’s protocol (IHC WORLD). The Nissl substance (rough endoplasmic reticulum) appears dark blue due to the staining of ribosomal RNA, giving the cytoplasm a mottled appearance. These features disappear in dead neurons. The surround glia cells (small size, weak-stained) were not counted. Nissl positive pyramidal cells (big size, strong-stained) were counted in a 250 × 250 μm square applied at the center of the CA1 and CA3 regions. Three consecutive sections per brain were analyzed and provided a single value for each mouse. The data were shown as the normalized percentages. FJc staining was performed as described in the literature (Schmued et al., 1997, 2005; Hopkins et al., 2000; Liang et al., 2008), brain sections were mounted with distilled water onto gelatin coated slides and dried at 50°C on slide warmer for 30 min. After rehydration in 100% ethanol for 5 min, 70% ethanol for 2 min, and distilled water for 2 min, the sections were oxidized in freshly prepared 0.06% potassium permanganate for 15 min followed with rinsing with distilled water for 2 min. The sections were immersed in 0.001% FJc dissolved in 0.1% acetic acid vehicle for 10 min and then rinsed three times with distilled water for 1 min. Thereafter, slides were air dried, cleared with xylene and coverslipped. The FJc positive cells of the CA1 and CA3 sub-regions of hippocampus were counted with ImageJ software in each of the three sections per animal. The average of the FJc positive cells (%) was expressed as percentage of the control. An observer blind to genotype and treatment performed cell counting.

### Western Blotting

At 24 h after administration of KA (20 mg/kg) or saline, animals were anesthetized and sacrificed by cervical dislocation. The hippocampus tissue was harvested immediately and stored at −80°C. For tissue lysate preparation, it was homogenized in RIPA buffer. The samples were centrifuged at 12,000 rpm for 5 min and supernatant was collected and kept on ice. Protein concentration was determined using the protein assay kit from Bio-Rad. Supernatant was separated on 4–15% gradient SDS-PAGE gels and transferred to nitrocellulose membranes. The integrated optic density of the immunoreactive bands was measured and quantified with ImageJ (National Institutes of Health). Primary antibodies used in this study include rabbit polyclonal anti-SIRT5 antibody (cell signaling, 1:1000), rabbit polyclonal anti-GPx antibody (Abcam, 1:1000), rabbit polyclonal anti-SOD2 antibody (Abcam, 1:2000) and mouse anti-actin (Millipore, 1:5000). Detection was carried out using horseradish peroxidase-conjugated anti-mouse, anti-rabbit antibody (GE Healthcare, 1:2000) and Thermo Scientific Pierce ECL Western Blotting Substrate.

### Quantitative Real-Time PCR

At 24 h after KA (20 mg/kg) or saline injection, tissue from hippocampus was dissected as described above from SIRT5^−/−^ and WT mice. Total RNA was extracted using Trizol (Invitrogen) and purified using a RNeasy kit (Qiagen) according to the Qiagen protocol. After isolation, RNA (20 ng per sample) was reverse transcribed to cDNA using SuperScript III RT Reverse Transcriptase (Invitrogen), which was stored at −20°C until further analysis. The mRNA levels were quantified with a quantitative real-time PCR (qPCR) system (Stratagene Mx4000). Each sample was detected in triplicate and each experiment was repeated three times. The following primers for expression analysis were designed using TaqMan assays (Life Technologies) and NCBI Primer-Blast: (5′→3′): SIRT5, forward, GCAGACGGGTTGTGGTCAT; reverse, CGATGCAACTCGTCAATGTTCT.

### Statistical Analysis

All quantitative data were performed using Prism software (GraphPad Software). The number of independent experiments is stated in the figure or figure legend. Survival was assessed using Kaplan–Meier analysis, and the statistically significant difference between two groups was determined with the log-rank test. Data from seizure scoring were analyzed using two-way analysis of variance (ANOVA) for repeated measurements. Unless otherwise noted, the comparison only between the two groups was analyzed using unpaired Student’s *t* tests, while multigroup comparisons were carried out using two-way ANOVA followed by Bonferroni *post hoc* tests. Results are expressed as group mean ± SEM and statistical significance was set at *p* < 0.05.

## Results

### SIRT5 Deficiency Increases Mortality Rate and Response to KA-Induced Seizures

**Figure 1:**
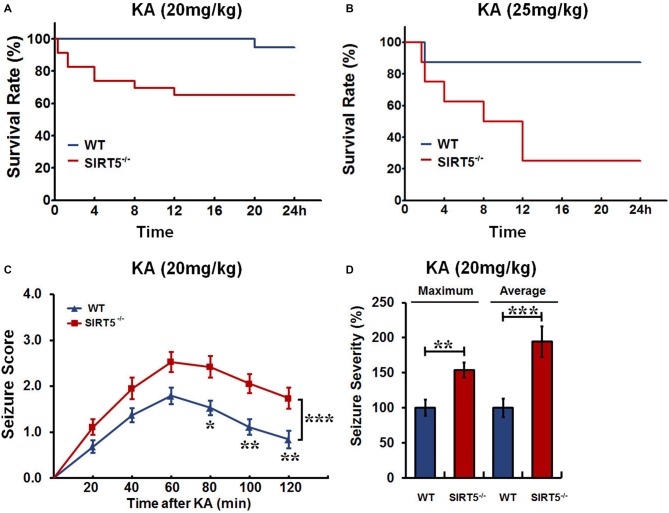
**Sirtuin 5 (SIRT5) deficiency increases mortality and seizure severity.** Kaplan–Meier survival curves show statistically significant difference (*P* < 0.05) in survival between SIRT5^−/−^ and control mice over 24 h following kainate (KA) injection at the dosage of both 20 mg/kg **(A)** and 25 mg/kg **(B)**. KA (20 mg/kg): SIRT5^−/−^ : *n* = 23, WT: *n* = 19; KA (25 mg/kg): SIRT5^−/−^ : *n* = 8, WT: *n* = 8. Survival was assessed using Kaplan–Meier analysis, and the statistically significant difference between two groups was determined with the log-rank test. **(C)** Seizure response over time in SIRT5^−/−^ (*n* = 19) and WT (*n* = 19) mice following KA injection (20 mg/kg). For each 20-min interval, the highest level of seizure activity was scored using seizure scale. With time, seizures were more severe in SIRT5^−/−^ mice. Data from seizure scoring were analyzed using two-way analysis of variance (ANOVA) for repeated measurements. **(D)** Integrated seizure severity of the cohort of mice. SIRT5^−/−^ : *n* = 23, WT: *n* = 19. The comparison was analyzed using unpaired Student’s *t* tests. Data in **(C,D)** are presented as mean ± SEM. *p < 0.05; **p < 0.01; ***p < 0.001; n.s., non-significant.

We first investigated whether SIRT5 affects seizure susceptibility. SIRT5^−/−^ and WT mice were injected with different dosages of KA, and the mortality rate and seizure severity were examined. The Kaplan–Meier survival curves showed that SIRT5 deficiency significantly increased the mortality (*p* < 0.05) in 24 h following KA injection at both lower (20 mg/kg) and higher (25 mg/kg) dosages (Figures 1A,B). At lower dosage, KA administration caused enhanced mortality by ~35% of SIRT5^−/−^ mice (*n* = 19), in contrast to less than 10% mortality rate of WT mice (*n* = 23, Figure 1A). Surprisingly, the mortality of SIRT5^−/−^ mice rose to 75% (*n* = 8) during 24 h following KA administration at higher dosage compared to 12.5% for WT control mice (*n* = 8, Figure 1B), further emphasizing the importance of SIRT5 and its regulation in this process.

To learn whether SIRT5 deficiency affects behavioral seizures, the seizure responses to KA (20 mg/kg) in SIRT5^−/−^ mice and control mice were evaluated by trained experimenter blinded to genotype according to reference descriptions (Ben-Ari et al., 1981; Yang et al., 1997). During 2 h after KA injection, mice of both genotypes developed acute, yet transient seizures that peaked between 40–80 min after KA injection and then decayed slowly (Figure 1C). With time, the majority of SIRT5^−/−^ mice underwent much more severe seizures (*p* < 0.001), whereas WT mice were less likely to suffer from seizures. In addition to seizure scores, seizure severity was calculated by integrating individual scores of each mouse over the experiment period (Giménez-Cassina et al., 2012). This method incorporates the seizure severity of mice that died in 2 h after KA injection, which can better reflect the whole picture. In contrast, SIRT5^−/−^ mice exhibited dramatic increase in both maximum (*p* < 0.01) and average (*p* < 0.001) seizure severity (Figure 1D). Together, our findings indicate that SIRT5 deficiency strikingly increases susceptibility to KA-induced epileptic seizures by using different dosages of KA injection and different analyses of seizure severity. Based on these results, mice treated with KA (20 mg/kg) or Saline were used for the following experiments.

### SIRT5 Deficiency Increases KA-Induced Hippocampal Neurodegeneration

**Figure 2:**
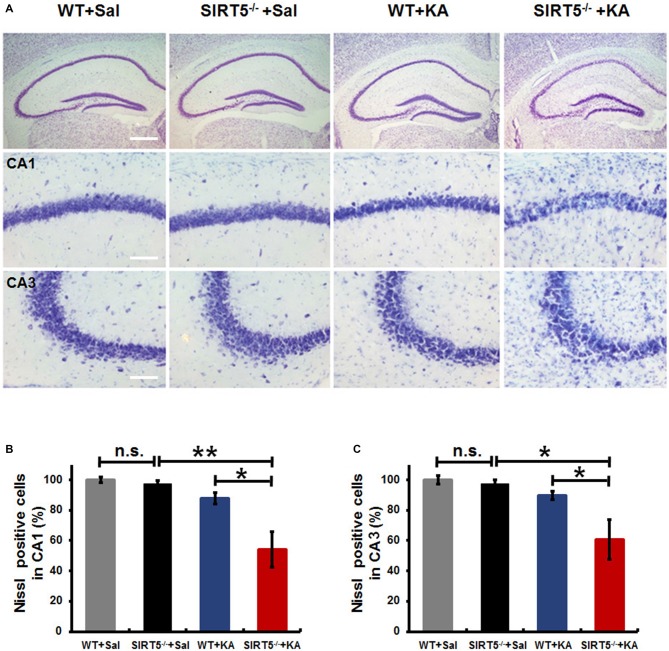
**KA-induced hippocampal neuronal death and degeneration is dramatically reduced in SIRT5^−/−^ mice.** All hippocampal slices were collected from WT and SIRT5^−/−^ mice 5 days after KA administration, and Nissl staining was performed for neuronal damage evaluation. **(A)** Representative hippocampus, CA1 and CA3 images from WT+Sal, SIRT5^−/−^+Sal, WT+KA, SIRT5^−/−^+KA animals. **(B)** Quantification was performed by counting the number of Nissl positive neurons (Sal groups: *n* = 4; KA groups: *n* = 6) in CA1 **(B)** and CA3 **(C)** regions. The data were carried out using two-way ANOVA followed by Bonferroni *post hoc* tests. Data are presented as mean ± SEM. *p < 0.05; **p < 0.01; n.s., non-significant; Sal, saline; KA, Kainate; WT, wild-type. Scale bar, 500 μm (**A**, top panel) and 100 μm (**A**, in hippocampal CA1 and CA3 regions).

**Figure 3:**
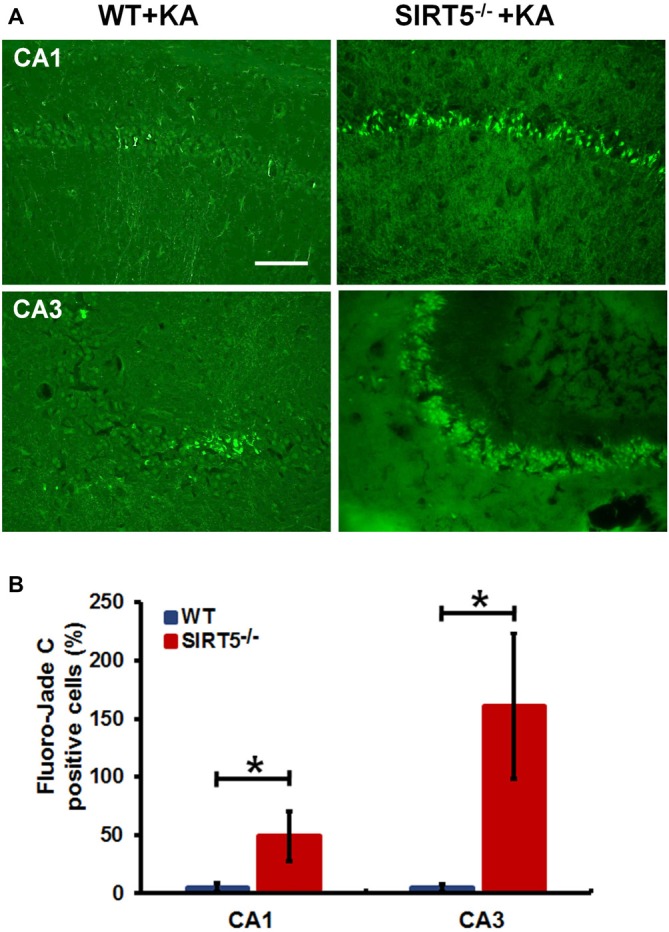
**SIRT5 deficiency exacerbates neurodegeneration in the hippocampal CA1 and CA3 regions after KA administration. (A)** Representative sections of Fluoro-Jade C (FJc) staining in hippocampus of WT and SIRT5^−/−^ mice are shown 5 days after KA administration. Significantly increased FJc-positive neurons were detected in the CA1 and CA3 regions of SIRT5^−/−^ mice compared to WT controls. **(B)** Quantitative analysis of FJc fluorescence in hippocampal subregions 5 days after KA (*n* = 6 rats per group). The comparison was analyzed using unpaired Student’s *t* tests. Data are presented as mean ± SEM. *p < 0.05. Scale bar, 50 μm.

KA-induced seizures are often associated with complex histological damages in hippocampus and other cerebral areas. In the hippocampus, delayed neuronal cell death of the CA1 and CA3 pyramidal neurons becomes apparent until 3–5 days in mice after KA injection (Kinoshita et al., 2012). To determine the role of SIRT5 in KA-induced neurodegeneration and whether SIRT5 deficiency may add this vulnerability, hippocampal neuronal death and degeneration of SIRT5^−/−^ and WT mice at 5 days after KA exposure, including those with similar seizure scores, were examined by Nissl staining and FJc staining of nearby sections (Figures 2, 3).

As shown in Figure 2A, under normal condition, no gross structural abnormalities were detected in SIRT5^−/−^ mice compared with WT control mice in all hippocampal regions. Severe neuronal death was observed in the hippocampal CA1 (54.2 ± 11.8%, 95 ± 21) and CA3 regions (60.5 ± 13.1%, 131 ± 29) of SIRT5^−/−^ mice at 5 days after KA administration, whereas WT mice exhibited comparably much less neuronal damage (CA1: 87.9 ± 3.7%, 155 ± 7; CA3: 89.7 ± 2.9%, 196 ± 6), which indicated that SIRT5 deficiency caused remarkably more severe hippocampal neuron degeneration compared with WT controls (*p* < 0.05, Figures 2A–C). Interestingly, no apparent neuronal loss was observed in dentate gyrus (DG) region in both genotypes, which was consistent with the previous report concerning the affected brain areas in rodent seizure model triggered by systemic KA administration (Wang et al., 2005). These morphological findings suggested neuroprotective effect of SIRT5 against KA-induced delayed cell death in hippocampus.

To further confirm the above assessment of severity of hippocampal neurodegeneration, we stained dying neurons in the hippocampus with FJc. Figure 3 shows the representative images of FJc staining in the hippocampus of SIRT5^−/−^ and WT control brains 5 days after KA administration. As expected, the FJc positive cells are predominantly confined to the pyramidal cell layers of the CA1 and CA3 regions, which are consistent with neuronal loss by Nissl staining. The dentate granule layer was not stained at all (not shown). This pattern of FJc staining is also consistent with that of neuronal death in hippocampus. In both CA1 and CA3, the average numbers of FJc positive cells in SIRT5^−/−^ group are significantly increased than the value for the WT control group (*p* < 0.05).

### SIRT 5 Deficiency Exacerbates Reactive Astrogliosis in Hippocampus

**Figure 4:**
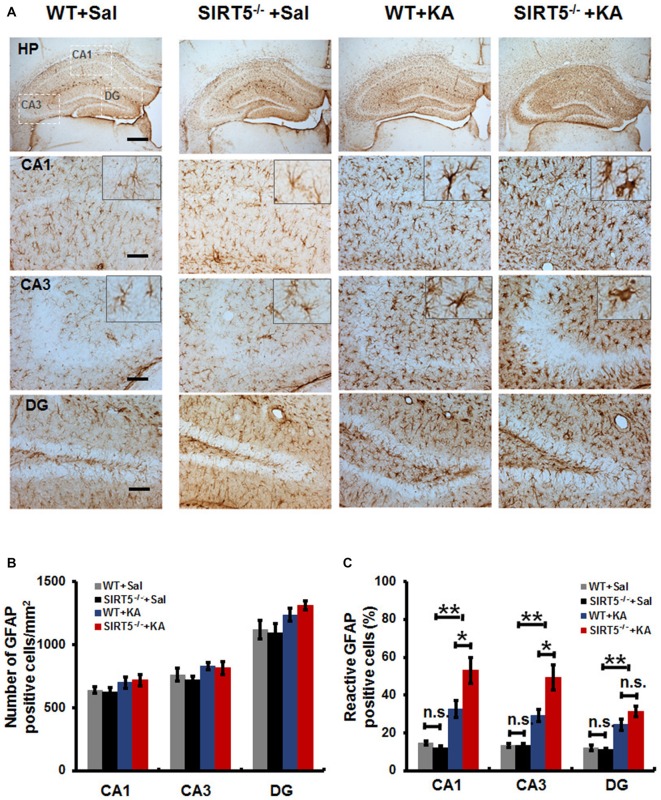
**SIRT5 deficiency exacerbates reactive astrogliosis in the hippocampus of mice after KA administration. (A)** Compared to nonreactive astrocytes in the control hippocampus, many astrocytes in the hippocampal CA1 and CA3 regions of KA administered mice were reactive and showed greater glial fibrillary acidic protein (GFAP) immunoreactivity, especially in SIRT5^−/−^ mice. Interestingly, the astrocyte reactivation in hippocampal dentate gyrus (DG) is not dramatically altered, comparable to that in the CA1 and CA3 regions. Quantification of the **(B)** total numbers of GFAP positive astrocytes and **(C)** the percentages of reactive astrocytes in different regions of whole hippocampus (*n* = 6 per group). The data were carried out using two-way ANOVA followed by Bonferroni *post hoc* tests. Data are presented as mean ± SEM. *p < 0.05, **p < 0.01. n.s., non-significant. Scale bar, 500 μm (**A**, top panel) and 100 μm (**A**, in hippocampal CA1, CA3 and DG regions).

Reactive gliosis, the morphological and biochemical changes in astrocytes, is often a prominent feature of human epilepsy and most animal models of recurrent seizures. To evaluate the effect of SIRT5 deficiency on hippocampal reactive astrogliosis following KA-induced injury, we investigated the phenotypes of astrocyte by immunostaining of GFAP, an astrocyte marker. In the normal condition (Figure 4A, left panels), astrocytes contiguously tiled the entire hippocampus and exhibited a regular distribution pattern in WT mice, most of which showed nonreactive condition (resting astrocyte) with small soma and 3–6 fine processes. No gross alteration was detected in SIRT5^−/−^ mice. Immunohistochemical analysis showed that KA exposure elicited a dramatic increase of GFAP immunoreactivity in the number and morphology change throughout the hippocampus at 5 days after KA administration, particularly prominent in the CA1 and CA3 areas; most astrocytes showed reactive phenotype characterized by hypertrophic cell bodies and intensely stained processes. SIRT5 deficiency apparently exacerbated KA-induced reactive astrogliosis (Figure 4A, right panels) in the CA1 and CA3 regions but not in the DG region. In addition, a few degenerated astrocytes with breakdown cell bodies were found in SIRT5^−/−^ mice but not in WT mice. Interestingly, KA exposure triggered apparent loss of astrocytes in the CA3 pyramidal layer, especially in SIRT5^−/−^ mice, which is consistent with previous report (Gualtieri et al., 2012).

Quantitative analysis showed that there was a slight increase in the total numbers of GFAP positive astrocytes in hippocampal CA1, CA3 and DG regions in both SIRT5^−/−^ and WT mice (Figure 4B, *P* > 0.05). Notably, a much higher percentage of reactive astrocytes induced by KA exposure was detected in the hippocampus, especially that was more prominent in the CA1 and CA3 regions in SIR5^−/−^ mice compared to WT controls (Figure 4C, *P* < 0.05). In order to avoid potential bias, two independent analyses were performed by two trained observers blinded to genotype and treatment. In summary, these results indicate that SIRT5 deficiency exacerbates KA-induced reactive astrogliosis in the hippocampus.

### KA Exposure Results in the Increased Expression of SIRT5 in Hippocampus

**Figure 5:**
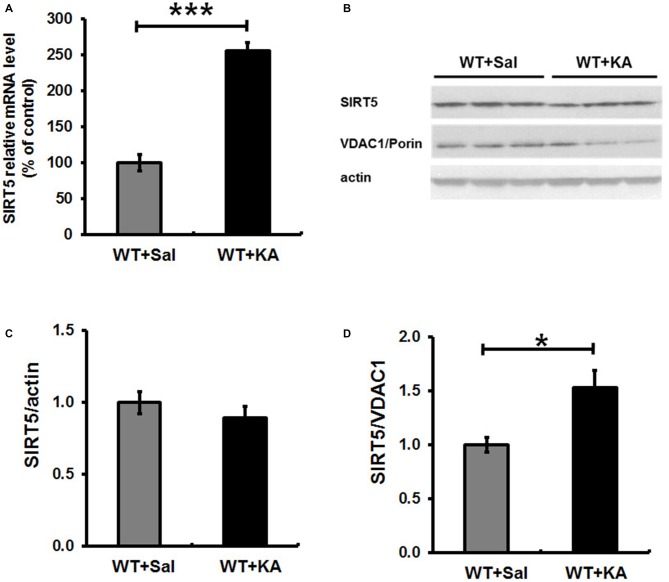
**Expression level of SIRT5 in hippocampus in 24 h following KA administration.** SIRT 5 expression levels were assessed by quantified with a quantitative real-time PCR (qPCR) and western blot. **(A)** SIRT 5 mRNA is dramatically upregulated in hippocampus of WT mice in 24 h after KA exposure. mRNA levels are expressed as a ratio to 18 S rRNA expression and normalized to baseline controls. Mean ± SEM, *n* = 4–5 per group. ***p < 0.001. **(B)** SIRT5 protein expression levels in 24 h after KA exposure. *n* = 4–5 per group. Although the total SIRT5 protein level was not notably altered **(C)**, the ratio of SIRT5 protein to VDAC1, a mitochondrial marker, was significantly increased 24 h after KA exposure **(D)**. The comparison was analyzed using unpaired Student’s *t* tests. Mean ± SEM, *n* = 4–5 per group. *p < 0.05.

SIRT5 expression is essential for maintaining proper mitochondrial function, which is required for metabolic homeostasis and regulation of the activity of multiple metabolic enzymes (Wallace, 2005). Next, we evaluated the impact of KA exposure on hippocampal SIRT5 expression. The mRNA and protein levels of SIRT5 were measured at 24 h following KA exposure (Figure 5). QPCR showed that SIRT5 mRNA expression level was more than 2.5 folds higher in 24 h after KA exposure (Figure 5A). Interestingly, Western Blot results demonstrated that the ratio of SIRT5 protein to actin was not obviously altered, but the relative ratio of SIRT5 protein to VDAC1/Porin, an outer membrane mitochondrial protein as mitochondria loading control, was significantly increased at 24 h after KA exposure (Figures 5B–D).

### SIRT5 Deficiency does not Affect the Decline of Mitochondrial ROS Scavenging Capacity in Hippocampus Damaged by KA

**Figure 6:**
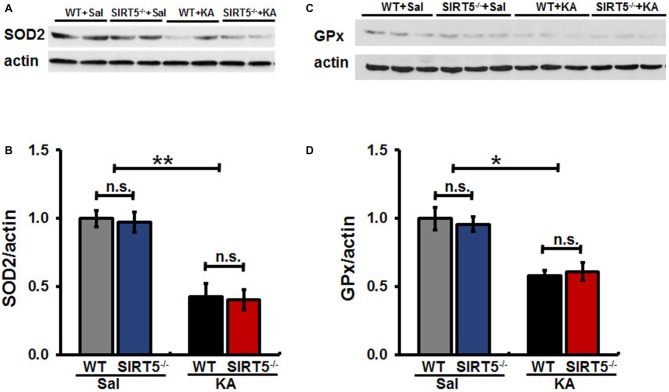
**SIRT5 deficiency does not affect the reduction of SOD2 and GPx proteins expression levels derived by KA exposure.** The effect of SIRT5 deficiency on protein expression levels of SOD2 and GPx induced by KA exposure: representative Western blot image showing decreased protein levels of SOD2 **(A)** and GPx **(C)** in 24 h in the hippocampus of SIRT5^−/−^ and WT mice exposure to KA. Densitometry analysis of SOD2 and GPx are shown in **(B,D)**. The data were carried out using two-way ANOVA followed by Bonferroni *post hoc* tests. Data are expressed as means ± SEM from *n* = 4–5 per group. *p < 0.05, **p < 0.01. n.s., non-significant. SOD2, superoxide dismutase 2; GPx, glutathione peroxidase.

Mitochondrial oxidative stress is emerging as a key factor that not only results from seizures, but might also contribute to epileptogenesis. To evaluate whether SIRT5 deficiency exacerbating KA-induced behavioral seizures and degeneration is associated with antioxidant capacity, we examined the expression levels of SOD2 and GPx in the hippocampus of mice by Western Blot (Figure 6). Our results showed that hippocampal SOD2 and GPx expression levels remarkably decreased at 24 h after KA exposure in both SIRT5^−/−^ and WT mice. However, to our surprise, at this time point in the present study, the declines of both enzymes triggered by KA were not significantly affected by SIRT5 deficiency. These data revealed that SIRT5 might contribute very little to preserve mitochondrial antioxidant capacity via SOD2 and GPx from oxidative injury caused by KA.

## Discussion

In rodents, systemic administration of KA can elicit sustained epileptic activity in the hippocampus. The animals exhibit recurrent behavioral seizures and progressive neuronal death, which provides a well-characterized model of neurodegenerative diseases (Wang et al., 2005; Shin et al., 2011; Zhang and Zhu, 2011; Zheng et al., 2011; Curia et al., 2014). The neurodegeneration occurs in adult limbic circuit, particularly in the CA1 and CA3 areas of hippocampus in rodents following systemic injection of KA, and this neuronal vulnerability is related to the selective distribution of the glutamate receptors (AMPARs/KARs) in hippocampus (Vincent and Mulle, 2009; Zheng et al., 2011; Kinoshita et al., 2012). Glutamate excitotoxicity has close synergistic interactions with oxidative stress and mitochondrial dysfunction, which contributes to KA-induced seizure and neuronal impairment and death in vulnerable brain regions (Novelli et al., 1988). Increasing evidences indicate that the ROS overproduction-induced oxidative stress results from glutamate medicated excitotoxicity (Löscher, 2002; Wang et al., 2005; Giardina and Gasior, 2009). Astrocytic activation and proliferation is the other characteristic of KA-induced brain damage, which is prominent in epilepsy.

Mitochondrial SIRT5 has been considered as an important functional modulator of mitochondria, which contribute to ageing and neurodegenerative diseases (He et al., 2012; Sack and Finkel, 2012; Osborne et al., 2014). Previous studies indicated that SIRT5 can substantially modulate neuronal excitation (Rardin et al., 2013; Buler et al., 2014; Kumar and Lombard, 2015; Polletta et al., 2015). This led us to hypothesize that SIRT5 may also influence neuronal excitability and consequently, the severity of seizures induced by chemoconvulsants. To test this hypothesis, we used the chemical convulsant agent KA to induce acute seizures via activating excitatory glutamate receptors (Contractor et al., 2011). The lower dosage of KA (20 mg/kg) was selected for the experiments to avoid ceiling and floor effects. We observed that the severity of KA-induced seizure was remarkably exacerbated in SIRT5^−/−^ mice compared to WT controls, which was confirmed by using two different quantification methods. Significantly, SIRT5^−/−^ mice exhibited much higher mortality rate to systemic injection of KA in a dosage-dependent manner, indicating the possible role of SIRT5 and its regulation in the process of epilepsy. The spontaneous recurrent seizures are associated with a transient abnormal hypersynchronous neuronal discharge. Neuronal electrical activity is substantially integrated with mitochondrial energy metabolism and redox balance (Zsurka and Kunz, 2015). Our findings demonstrated that lack of SIRT5 greatly exacerbated KA-induced seizure severity and mortality of mice, supporting the protective role of SIRT5 in this seizure model. It might present a potential target for seizure suppression in multiple clinical settings. In addition, it is noteworthy that SRIT5-null mice are viable and fertile, and lack gross physiological defects (Nakagawa et al., 2009). Therefore, pharmacological inhibition of SIRT5 is unlikely to cause obvious abnormality.

Recurrent seizures are often accompanied by neuronal loss in human patients and animal models (Chang and Lowenstein, 2003; Curia et al., 2014). The severity of neural damage is also a key factor in determining the mortality and morbidity under epilepsy and other pathological conditions. It is well documented that during acquired epilepsy, structural change or lesions in the brain including neuronal loss and marked reactive astrogliosis are often visible. Neuropathological investigations have repeatedly described a similarity between seizure and related neuronal alterations characterized by swollen and disrupted mitochondria. Systemic injection of KA produced extensive neuronal death, primarily within CA3 and CA1 areas. The delayed cell death in CA1 appears to be more apoptotic compared to acute, primarily necrotic neurodegeneration of CA3 and hilar neurons (Gualtieri et al., 2012). Nissl staining has been widely used as a neuronal death marker. This method stains Nissl substance in the cytoplasm of neurons, and is used to highlight important structural features of neurons. The Nissl substance (rough endoplasmic reticulum) appears dark blue due to the staining of ribosomal RNA, giving the cytoplasm a mottled appearance. These features disappear in dead neurons. As such, we selected Nissl and FJc staining as the pyramidal neuronal death and degeneration markers to test the effect of SIRT5 deficiency on KA triggered neurodegeneration in hippocampal CA1 and CA3 regions. In the present study, systemic administration of KA produced well-characterized seizures and selectively hippocampal neurodegeneration, which was consistent with previous report (Wang et al., 2005; Lee et al., 2010). Hippocampal pyramidal neurons are singularly vulnerable to activity dependent cell death following excitotoxic insults triggered by KA. Previous reports have demonstrated that FJc, an anionic fluorescein derivative, is a very sensitive histological staining marker evaluating undergoing degeneration of cell bodies, axons, dendrites, or terminals (Schmued et al., 2005). Our results provide compelling evidence that the two hippocampal subregions showed different vulnerability profiles to KA-triggered injury. We found that lack of SIRT5 dramatically exacerbated KA-induced neurodegeneration in the hippocampal CA1 and CA3 regions, indicating the potential neuroprotective role of SIRT5 in KA-induced seizure mice models. The stimulation of KA receptors directly excites CA3 neurons and increases glutamate efflux. It was reported that the mechanisms underlying glutamate receptors and consequent Ca^2+^-mediated excitotoxicity mainly involve activation of proteases, mitochondrial Ca^2+^ overload, excessive ROS production, and reduced ATP production, all of which act cooperatively to damage membranes, cellular proteins, DNA and mitochondrial function, eventually leading to necrosis, apoptosis or both (Rossi et al., 2007; Khurana et al., 2013). SIRT5 deficiency may contribute to mitochondrial metabolic dysfunction and the deficit of mitochondrial antioxidant system.

In healthy brain, astrocytes are organized in a very particular manner. They send out many fine branches covering synapses and enwrapping adjacent brain capillaries residing within their anatomy domains. The functions of astrocytes involve potassium buffering, interstitial volume regulation, a low interstitial glutamate concentration control and synchronize neuronal firing. Astrocytes response to central nervous system (CNS) damage through reactive astrogliosis, that involves structural and metabolic changes. Reactive astrogliosis has been considered as a prominent feature of mesial temporal lobe human epilepsy and most animal models of recurrent seizures (Wetherington et al., 2008; Curia et al., 2014; Robel and Sontheimer, 2015; Sofroniew, 2015; Wilcox et al., 2015; Pekny et al., 2016). The proliferative response of astrocytes induced by KA exposure has been reported in 1982 (Murabe et al., 1982), and exhibits a steady increase from at least 1 or 3 days to 1 month after KA exposure (Bendotti et al., 2000; Ding et al., 2000). In our study, the normal organization of astrocytic domain was disrupted in mice exposed to KA, as revealed by GFAP staining. These reactive astrocytes exhibited hypertrophic cell bodies and thick processes. The abnormal ratio of astrocytic volume in the whole hippocampus was more severe in SIRT5^−/−^ mice exposed to KA, accompanied by more severe neurodegeneration in hippocampal CA1 and CA3 regions. In epileptic tissues, reactive astrocytes may be involved in seizure development via a variety of specific mechanisms (Wetherington et al., 2008; Robel and Sontheimer, 2015). The morphological changes occurred during reactive astrogliosis are paralleled by functional changes. Human and animal studies have shown the downregulated glutamate transporter GLT-1 in reactive astrocytes, as well as glutamine synthetase in epilepsy tissues (Maragakis and Rothstein, 2004). This may result in an increase of glutamate in astrocytes leading to its excessive release, triggering synchronized depolarization of neurons. Reactive astrocytes also exhibit downregulated inward rectifying K^+^ channels in temporal lobe epilepsy, thereby leading to impairment of extracellular K^+^ clearance, which is linked to hyperexcitability of neuron (Oberheim et al., 2008; Sofroniew, 2009). Additionally, the major water channel (aquaporin-4) loses its polarized location in endfeet of astrocyte and becomes distributed across the entire cell, potentially leading to imbalances of both water homeostasis and K^+^ buffering (Binder et al., 2012; Nagelhus and Ottersen, 2013; Coulter and Steinhäuser, 2015). Since the reactive astrogliosis was observed 5 days after KA exposure, the activated astrocyte might contribute to inflammation that could further promote neuronal cell loss (Vinet et al., 2016). Together, the data indicates that SIRT5 deficiency may affect the functional deficit in reactive astrocytes that play critical roles in regulating seizure susceptibility and affecting neurodegeneration.

Brain injury caused by seizures and epilepsy is a dynamic process that comprises multiple factors contributing to neuronal cell death. Mitochondrial dysfunction and oxidative stress contribute to neuronal death, which is the key feature of neurodegeneration (Rowley et al., 2015). KA exposure enhanced SIRT5 mRNA expression level and relatively increased its protein level in survived mitochondria, which might contribute to the above beneficial effects against the damage in hippocampus. It will be more informative to indicate the respective cellular SIRT5 expression level (neuronal, astrocyte and microglia, etc.) in the brain. But currently it is not feasible since there is no excellent commercial SIRT5 antibody that can be used for double-immunostaining with neuron or glia markers. We were considering why the KA exposure only affected the mitochondrial SIRT5 protein expression relatively but not total protein level reflected by the ratio of Sirtuin5/actin. Since mitochondria are the primary site of ROS production that makes them particularly vulnerable to oxidative stress damage, KA exposure induced injury eventually resulted in mitochondrial degeneration and loss, which might cover up the increase of SIRT5 expression triggered by KA exposure. Systemic KA causes a series of progressively severe and highly stereotyped behavioral seizures that originate in the hippocampus, which in turn increases mitochondrial oxidative damage (Lothman and Collins, 1981; Zeng et al., 2007). Previous studies showed that SOD2 deficent mice (Sod2^−/+^) exhibited exacerbated KA-induced mitochondrial aconitase inactivation and neuronal loss in hippocampus (Liang and Patel, 2004), and overexpression of SOD2 in mice attenuated both of these changes, although KA-induced dramatic reduction of SOD2 and GPx were observed, we did not find the apparent effect of SIRT5 deficiency on the decline of these antioxidative enzymes under our paradigm.

The effect of mitochondria in acquired epilepsies, which account for about 60% of all epilepsies, is important but less well understood. Although mitochondrial oxidative stress and dysfunction are associated with inherited epilepsies, little is known concerning their contributions in acquired epilepsies. Initial trauma of KA exposure triggers complex cellular and molecular events, leading to functional and structural modification in the brain, which contributes to the pathogenesis of epilepsy and, as a result, recurrent seizures. Both functional and pathological defects of mitochondria have been reported in epileptogenesis (Volmering et al., 2016). Further investigations are needed to encode the natural property of SIRT5 in mitochondria and their contribution in epilepsy and neurodegeneration.

In conclusion, the present study shows that SIRT5 deficiency strikingly increased mortality rate and seizure severity, exacerbated hippocampal neuronal death and degeneration, and triggered more severe reactive astrogliosis in mice exposed to KA. We found that the expression of mitochondrial SIRT5 of injured hippocampus was relatively up-regulated, indicating its potential contribution to the comparably increased survival of these cells and its possible neuroprotective role. SIRT5, to our surprise, did not seem to apparently alter the decline of antioxidant enzymes SOD2 and GPx in hippocampus caused by KA exposure in our paradigm. The protective mechanism of SIRT5 on seizures and cellular degeneration might through different regulatory mechanism other than antioxidant enzymes SOD2 and GPx, which we would like to explore in the future experiment. For the first time we illustrate the role of SIRT5 in KA-induced mice model, offering a new understanding of roles of SIRT5 in mitochondrial functional regulation and their potential neuroprotection. These results will enrich our acknowledgment of the natural property of SIRT5 in health and neurological diseases.

## Author Contributions

All authors have made substantial, intellectual and direct contribution to the work, and approved it for publication.

## Conflict of Interest Statement

The authors declare that the research was conducted in the absence of any commercial or financial relationships that could be construed as a potential conflict of interest.
